# Learning on Lockdown: A Study on Medical Student Wellness, Coping Mechanisms and Motivation during the COVID-19 Pandemic

**DOI:** 10.15694/mep.2021.000050.1

**Published:** 2021-02-17

**Authors:** Lori Deshetler, Megha Gangadhar, Dhanushya Battepati, Erin Koffman, Ram Kishore Mukherjee, Bindu Menon

**Affiliations:** 1 University of Toledo College of Medicine and Life Sciences; 2 University of Toledo

**Keywords:** COVID-19, pandemic, medical student wellness, medical education

## Abstract

This article was migrated. The article was marked as recommended.

**Background:** The COVID-19 pandemic forced drastic changes to the educational settings and living conditions of medical students nationwide. Due to the sudden onset of remote learning, drastic educational changes likely induced fear and anxiety among students. Therefore, the purpose of this study was to assess medical students’ academic and personal wellbeing, coping mechanisms, and motivation during the Stay at Home order through a survey-based study.

**Methods:** The researchers obtained Institutional Review Board approval, and the study was classified as exempt category 2. In total, 705 medical students representing all four years were recruited from the researchers’ institution. The survey was administered through an anonymous link in Qualtrics to the medical student listserv, and responses were collected over a two-week period. The instrument was comprised of four demographic questions, twenty-two closed-ended questions, and four open-ended questions.

**Results:** A total of 188 medical students completed the survey for a response rate of 26.7%. Due to a very low response rate (n=3, 1.6%) from fourth-year students, quantitative data from this group were excluded. Quantitative analysis of the survey results showed that most respondents experienced nervousness and stress at some point during the pandemic. While most claimed to have successfully overcome challenges and achieved their goals, only about half of the participants admitted to being able to handle their personal problems, and nearly 70% of the respondents expressed difficulty focusing while studying. The qualitative data suggest that changes in study environment, long-term home confinement, and isolation were the most challenging aspects for participants.

**Conclusions:**Overall, this study showed that the COVID-19 pandemic induced stress and anxiety among students; nevertheless, most were able to employ effective coping strategies, pursue mental wellness assistance, achieve goals, and maintain motivation during the pandemic.

## Introduction

In December 2019, the first case of Coronavirus (COVID-19) was reported in Wuhan, China ( Fauci, Lane and Redfield, 2020). Within one month, the virus had spread across the world, including the Unites States ( Wu, Chen and Chan, 2020), infecting over six million individuals globally in the next 5 months. COVID-19 has overwhelmed hospital systems worldwide and devastated countless families, economies, and livelihoods. Consequently, drastic measures were adopted including Stay at Home orders, cancelled work for non-essential employees, and mandated remote learning for students, as an attempt to quell viral transmission ( Courtemanche *et al.*, 2020). Stringent quarantine measures were adopted in the United States soon ( Parmet and Sinha, 2020) after the World Health Organization declared COVID-19 to be a pandemic on March 11, 2020 ( Cucinotta and Vanelli, 2020). In Ohio, Governor Mike DeWine promptly reacted to the first three Ohio COVID-19 cases by declaring a state of emergency and enacting a Stay at Home order ( Dorn, Cooney and Sabin, 2020). In response, many educational institutions statewide, including The University of Toledo, immediately mandated remote learning for students ( Woolliscroft, 2020).

COVID-19 is a novel virus and despite a growing body of research ( Cao *et al.*, 2020; Chang, Yuan and Wang, 2020; Tang *et al.*, 2020), its emotional impact among student populations remains poorly understood. Specifically, medical student mental health and learning ( Ferrel and Ryan, 2020; Sierpina, 2020) during the pandemic remains largely unknown. The fear and uncertainty surrounding COVID-19 combined with social distancing measures have been shown to increase stress, anxiety, loneliness, and feelings of isolation ( Torales *et al.*, 2020). Since medical student wellness and burnout have been points of concern historically ( Hansell *et al.*, 2019), inadequate support resources (e.g., absence of in-person counseling facilities) could exacerbate existing mental health conditions. Aside from this, the reduction of in-person learning and patient interactions faced by students at many medical schools ( Ahmed, Allaf and Elghazaly, 2020) may have contributed to decreased motivation and wellness during the pandemic. The benefits of in-person and team-based learning on educational satisfaction is well documented ( Roh, Lee and Mennenga, 2014). Consequently, there is an impetus to explore whether remote learning, with less opportunity for student-to-student interaction, has decreased motivation to learn and wellness markers in students during COVID-19. The aim of this study was to assess how the COVID-19 quarantine and mandated remote learning influenced wellness, coping, and motivation among The University of Toledo College of Medicine and Life Sciences (UTCOMLS) medical students. We hypothesized that COVID-19 negatively affected medical students’ wellness due to increased stress and anxiety levels. Results of this study will provide the medical education community with objective data to analyze student responses during the pandemic.

## Methods

### Design

A descriptive, survey-based study was used to identify personal and academic wellness, stress, motivation, and coping mechanisms employed by UTCOMLS medical students during the COVID-19 pandemic. In total, 705 students from all four years were recruited into the study during June 2020. Responses were collected over a two-week period, and email reminders were sent to increase the response rate. The survey instrument (Supplementary File 1) consisted of six sections, including four demographic questions, twenty-two closed-ended questions, and four open-ended questions. The survey was administered via a Qualtrics survey link sent to the medical student listserv. All submissions were reported anonymously.

### Outcome Measures/Variables

The first of the six survey sections questioned respondents on four demographic factors, including graduating class, age, gender, and ethnicity. The remainder of the survey sections consisted of twenty-two closed-ended questions from well validated standardized instruments ( Cohen, Kamarck and Mermelstein, 1983; Chen, Gully and Eden, 2001; Sinclair and Wallston, 2004), some of which were modified along with the corresponding scale, and four open-ended questions related to wellness, perceived stress, coping mechanisms, and general comments.

Wellness levels were assessed using six questions from the General Wellness and Self Efficacy Survey (NGSE) ( Chen, Gully and Eden, 2001) with the following scale: Strongly disagree-Disagree-Neither agree nor disagree-Agree-Strongly agree; stress levels were assessed using five questions from the Perceived Stress Scale (PPS) ( Cohen, Kamarck and Mermelstein, 1983) with the following scale: Never-Rarely-Sometimes-Frequently-Always; finally, coping levels were assessed using five questions from the Brief Resilient Coping Scale (BRCS) ( Sinclair and Wallston, 2004) with the following scale: Strongly disagree-Disagree-Neither agree nor disagree-Agree-Strongly agree. Additionally, we included open-ended questions to capture students’ perceptions of their challenges, adjustment to the new learning environment, and methods used for coping during the pandemic.

### Statistical Analysis

Survey data were analyzed using Qualtrics. Examination of data included calculation of contingency tables and bar-charts for each question. The chi-square test was used to assess if different class, gender, and age were independent of the five choices for each question. We used the non-parametric Kruskal-Wallis test to analyze if there were any significant differences across class, gender, age group, or ethnicity. Upon finding a difference, a post-hoc test was carried out to determine which groups were significantly different. Nonparametric statistical analyses were completed using the Dunn’s test of multiple comparisons, and multinomial confidence interval tables were completed using DescTools of R-version R-3.4.4. Unless otherwise noted, all testing was two-tailed and evaluated at the Type I Error Rate of alpha=0.05 level of statistical significance. Since there are low counts in some cells, the chi-square approximation may not be appropriate. Hence, p-values generated from chi-square are simulated p-values.

The second part of the analysis included coding of the qualitative responses for themes. The researchers grouped common terms and ranked these from most to least cited. A frequency threshold of 20% was utilized for identifying themes in the open-ended responses.

### Ethical considerations

Confidentiality of personal information was maintained, and no identifiers were collected as part of the survey. Student participation in this survey was voluntary and uncompensated. Prior to starting the survey, all students were made aware of the purpose of the study and informed consent was acquired. Students were provided with the option to click “Disagree” when prompted for informed consent, in which the student was taken to the end of the survey. Students were made aware that the study was classified exempt by the Institutional Review Board of The University of Toledo.

## Results/Analysis

A total of 188 medical students completed the survey for a response rate of 26.7%. Of the respondents, 75 (39.9%) were male and 113 (60.1%) were female. The majority (n= 126, 67.0%) were between the ages of 24-31, and most (n=130, 66.0%) were White or Caucasian. Additionally, the number of completed surveys obtained from M1s, M2s, M3s, and M4s were 88 (50.3%), 56 (32.4%), 41 (23.4%), and 3 (1.6%), respectively.

The survey assessed general wellness and self-efficacy, perceived stress, coping abilities, and motivation during the COVID-19 Stay at Home order. Using the Binomial test, we found that a significant (p<0.05) number of respondents were able to achieve their academic goals. Compared to the rest of the cohorts, the proportion of participants in the M3 class were found to be significantly (p<0.05) different. However, we did not find any differences between age groups or gender. Similarly, the number of students who thought that “in general, I think that I obtained outcomes that are important to me” was significant (p<0.001). Students from the 24-31 age group agreed to this significantly (p<0.05) more than the 32-39 age group. On the item related to being able to “successfully overcome challenges,” a significant (p<0.05) number of students Strongly agreed/Agreed. While there were no major differences among the different demographics, we found that the 24-31 and 32-39 age groups were statistically (p<0.05) different, as more students from the younger age group agreed to obtaining desired outcomes. A significant (p< 0.001) number of students also agreed that they “performed effectively on many different tasks,” “compared to other people, completed most tasks very well,” “successfully overcame challenges,” and “even though things were tough, performed quite well.” However, in all categories, we found that the percentage of students who agreed were significantly (p<0.05) higher in the younger age group (24-31), compared to the older age group (32-39). Similarly, the M3 class performed significantly (p<0.05) worse than the M1 and M2 cohorts in the first two categories.

Regardless of students’ positive self-efficacy sentiments, more than half of respondents admitted to experiencing frequent “nervousness and stress” during the Stay at Home order. This was most significantly (p<0.05) seen among the older age group (32-39) and respondents who reported Asia/Pacific Islander ethnicity when compared to the rest. However, while a significant (p<0.05) number of students thought that they were “effectively coping with changes in their lives,” the number of students who thought they “were on top of things” was insignificant. There were no remarkable changes between the various demographics in their response to the question regarding “effectively coping with important changes that were occurring in your life;” however, significantly (p<0.05) more individuals in the younger age group (24-31) felt they were “on top of things.” Student stress levels were further evidenced by a significant (p<0.01) number of participants who reported that difficulties were piling up to an unsurmountable volume, which was again significantly (p<0.05) more observed among the older age group (32-39), compared to the 24-31 age group. Nevertheless, survey respondents from all demographics overwhelmingly expressed confidence in their ability to handle their personal problems (p<0.001).

A significant (p<0.001) number of survey respondents, across all demographics, unanimously reported the ability to effectively cope with the Stay at Home order. The majority stated they “effectively maintained balance in their lives,” “grew positively from difficult situations” presented, sought “creative coping strategies,” and “pursued mental wellness assistance” to cope with the pandemic. Therefore, despite frequent nervousness and stress, students were able to cope with challenges effectively and did not hesitate to seek help. The older age-group (32-39) sought mental assistance more significantly (p<0.01) than their younger classmates (24-31).

Student responses regarding their motivation during the pandemic showed that most were able to continue with their studies, despite reporting feelings of boredom when educational materials were dull; however, a significant (p<0.001) number of students indicated they struggled with distractions that affected their motivation. The responses to these questions also revealed that even with the challenges presented by the Stay at Home Order, students found ways to prepare for their future careers. Similarly, it is noteworthy that a significant (p<0.05) number of respondents from the older age group (32-39), despite being more “stressed and nervous” than the younger age-group (24-31), agreed that “when I needed help, I did not hesitate to seek assistance,” and “I sought assistance for mental wellness to cope with the pandemic.”

## Discussion

To our knowledge, this is the first study to investigate medical student wellness, motivation, and coping mechanisms during the COVID-19 pandemic in the United States. The pandemic has been stress-inducing for many individuals, and long-term psychological effects have been predicted by psychologists ( Rahman *et al.*, 2020). As COVID-19 cases continue to rise ( Li and Meng, 2020) in several states, including that of the researchers, and with experts warning that the pandemic may resurface more severely in the fall or winter months ( Rose, 2020), it is increasingly pertinent that educational leaders understand how the pandemic impacts students. The results of this study provide quantitative data to illustrate medical student wellness, coping, and motivation during the COVID-19 Stay at Home order.

The data collected display a trend among students-during the pandemic, most respondents were able to demonstrate overall academic success and resilience. Simultaneously, the majority of responding students also reported increased nervousness, stress, and distraction. It was noteworthy that altogether the older age-group (32-39) reported more stress and difficulty, and they also sought mental health assistance more significantly (p<0.01) compared to the younger group (24-31). Survey respondents reported that they experienced several challenges while adjusting to the new social distancing regulations and remote learning environment. Responses indicated that more than 156 (84%) of participants experienced nervousness and stress due to these changes. In fact, more than one-third stated that the most challenging aspect during the pandemic was due to their new learning environment. However, most respondents reported the ability to cope with the changes, manage their personal problems, and seek assistance for mental wellness. Notably, students at our institution were able to benefit from remote resources offered by the university and personal resources, such as family and friends. Although the benefits of in-person counseling are well known ( Devi *et al.*, 2013; Vidourek *et al.*, 2014), it can be inferred that most students were able to access alternate support systems (e.g., friends, family, and relatives). Understanding which resources were most beneficial for students will be extremely informative for administrators who will need to provide students with ongoing academic and emotional support in the coming year as the pandemic persists.

During the Stay at Home order, a vast majority of respondents displayed confidence in their work output despite increased stress levels. This was another surprising finding, as the adverse effects of unfavorable stress on academic performance is well known ( Kumar *et al.*, 2014; Alsaggaf *et al.*, 2016; Crego *et al.*, 2016; Kotter *et al.*, 2017). It is possible that students were able to dedicate more time to studying due to restrictions on extracurriculars and social settings. Interestingly, while students were able to claim self-efficacy on an individual basis, the majority were unable to rate their accomplishments in comparison to their peers. This points to the possibility that social distancing decreased peer-to-peer competition during the pandemic.

### Limitations

With the importance and urgency of this study, there are several limitations to consider. First, the scalability of responses may be limited as this study was only conducted with medical students from a single institution. Additionally, since only medical students were surveyed, the results may not directly translate to students in other educational programs. Also, although the survey was distributed to all four years of medical students, the breadth of responses was limited. Most submissions were from M1s during their summer vacation, while submissions from M3s and M4s were considerably less, most likely due to rigorous rotation schedules and preparation for residency. Interpretation of statistically significant results may have limited clinical value due to small sample sizes in some groups, such as the 32-39 age group. Being an observational study, one cannot discount the possibility that other confounding variables (e.g., demographic information) may have influenced some responses. Finally, analysis of the open-ended responses may have incorporated some level of subjectivity, as is typical with qualitative data coding. Irrespective of these limitations, this study provides The University of Toledo medical school leaders, as well as other educational administrators, with considerations for planning future remote learning experiences while mitigating any potentially adverse long-term educational and psychological implications.

## Conclusion

The study results showed that although stress and anxiety levels were increased, respondents were able to adequately cope with challenges and efficiently dedicate time to their studies. To the best of our knowledge, this is the first study of its kind to investigate the psychological and educational impacts of COVID-19 on medical students in America. As COVID-19 remains an imminent threat to communities nationwide, it is pivotal that medical education leadership understands the repercussions of remote learning on student wellness. The results of this study will enlighten administrators on how to best equip students with resources to ensure minimal educational and emotional disruption.

## Take Home Messages

• To alleviate COVID-19-induced stress and anxiety, it should be ensured that medical students have access to mental health services.
• Schools should coordinate support groups and provide subscriptions to apps for mental wellbeing.
• Safe, socially distanced options should be offered to medical students to promote interaction with peers to combat feelings of home confinement and isolation.
• Schools should provide more opportunities for students to maintain motivation to learn.

## Notes On Contributors

**Lori M. DeShetler**, Ph.D., is the Assistant Dean for Assessment and Accreditation in the Department of Medical Education at The University of Toledo. Dr. DeShetler’s current research interests include assessment, COVID-19 impact on medical students, curriculum mapping, and implications of the opioid crisis on medical education. ORCID ID: https://orcid.org/0000-0002-4566-7111

**Megha Gangadhar** is a second-year medical student at The University of Toledo College of Medicine. Ms. Gangadhar’s current research interests include COVID-19 impact on medical students and Ophthalmology case studies.

**Dhanushya Battepati**, MS, is a second-year medical student at The University of Toledo College of Medicine and Life Sciences. Ms. Battepati’s research interests include COVID-19 impact on medical students, global health disparities, and cardiovascular medicine.

**Erin E. Koffman**, MS, is a medical student at the University of Toledo. Her research interests include the neurobiology of mental health disorders, acid-sensing ion channels, preventative health, and COVID-19 impact on medical students. ORCID ID: https://orcid.org/0000-0002-5230-7499

**Ram Kishore Mukherjee**, MS, is a graduate student in the Department of Mathematics and Statistics at The University of Toledo. Ram’s current research interests include the change in the electrical activity of the diaphragm in response to painful procedures on neonates, COVID-19 impact on medical students, and Semi parametric inference on Numbers needed to treat. ORCID ID: https://orcid.org/0000-0001-7590-8900

**Bindu Menon**, Ph.D., is an Assistant Professor in the Department of Medical Education at The University of Toledo. Dr. Menon’s research interests include vertical integration of foundational science elements into clinical years, COVID-19 impact on medical students, cognitive assessment of student mastery in assessments, and analysis of NBME subject examination results. ORCID ID: https://orcid.org/0000-0002-4436-8208
