# Supplementary material for: Learning on Lockdown: A Study on Medical Student Wellness, Coping Mechanisms and Motivation during the COVID-19 Pandemic
Source: MedEdPublish (2016). 2021 Feb 17;10:50. [Version 1] doi: 10.15694/mep.2021.000050.1 (PMC10939617; doi:10.15694/mep.2021.000050.1)
Supplement: Peer review history

# Reviewer report: Reviewer response for version 1

**DOI:** 10.21956/mep.18915.r27036

**Published:** 2021-06-06

**Authors:** Ken Masters

**Affiliations:** 1 Sultan Qaboos University

This review has been migrated. The reviewer awarded 3 stars out of 5 An interesting paper on medical student wellness, coping mechanisms and motivation during the Covid-19 pandemic. The background identifies the need to understand student problems brought about by the pandemic, and the coping mechanisms employed. To understand these, the authors ran a survey among their students. The survey form design was based on other, similar, surveys and validated scales.While the response rate is rather low, the figures are still useful to gain some insight into the most important problems experienced by the students.There are a few issues in the paper that do need to be addressed.• The Abstract states: “Due to a very low response rate (n=3, 1.6%) from fourth-year students, quantitative data from this group were excluded.” This also needs to be given in the body of the paper.• The statistics are given in textual format, but this means that some are hidden, and also makes for difficult reading. As the survey was detailed, and would have received detailed responses to all other questions, these should all be shown in detailed tables. (A simple principle of “don’t gather data unless you use it” applies, so please show all of the results).• Examples of the qualitative should be formally reported, in their themes, with an indication of how the theming was performed (e.g. who performed, verified, software (if any) used, and why a 20% threshold was chosen). So, overall, a useful study, but I would like to see Version 2 of the paper that addresses these issues.

Reviewer Expertise:

NA

No decision status is available

# Reviewer report: Reviewer response for version 1

**DOI:** 10.21956/mep.18915.r27035

**Published:** 2021-02-23

**Authors:** BALAJI ARUMUGAM

**Affiliations:** 1 TAGORE MEDICAL COLLEGE AND HOSPITAL

This review has been migrated. The reviewer awarded 4 stars out of 5 Medical Student Wellness, Coping Mechanisms and Motivation during the COVID-19 Pandemic done among medical students using a standardized instrument scale with open ended questions also. Overall this study was done as a quantitative questionnaire based online survey. The study results showed that although stress and anxiety levels were increased, respondents were able to adequately cope with challenges and efficiently dedicate time to their studies. These type of studies to be done more with maximum sample size among medical students and also among the medical practitioners who are undergoing tremendous anxiety and stress during this COVID PANDEMIC. One of my suggestions or question to get clarified would be on the scale / survey instruments used from various sources from other published articles. Did the permission was sought for using the survey instruments for the research purpose? Please clarify? Thanks for inviting to review this article.

Reviewer Expertise:

NA

No decision status is available
